# Supplementary material for: Metal Cluster Triggered-Assembling Heterogeneous Au-Ag Nanoclusters with Highly Loading Performance and Biocompatible Capability
Source: Int J Mol Sci. 2022 Sep 23;23(19):11197. doi: 10.3390/ijms231911197 (PMC9569858; doi:10.3390/ijms231911197)
Supplement: Supplementary file 1 [file ijms-23-11197-s001.zip › ijms-1901110-supplementary.pdf]

Metal Cluster Triggered-Assembling Heterogeneous Au-Ag Nanoclusters with Highly Loading Performance and Biocompatible Capability

Xiaoxiao He[1, 2#], Yujun Yang[3#], Xiaohong Ma [4#], Xi Hu[1, 2], Teng Wang [1, 2], Shiyue Chen [1, 2], Xiang Mao [1, 2]*

1. State Key Laboratory of Ultrasound in Medicine and Engineering, College of Biomedical Engineering, Chongqing Medical University, Chongqing 400016, P. R. China

2. Chongqing Key Laboratory of Biomedical Engineering, College of Biomedical Engineering, Chongqing Medical University, Chongqing 400016, P. R. China

3. Key Laboratory of Laboratory Medical Diagnostics, Ministry of Education, Department of Laboratory Medicine, Chongqing Medical Laboratory Microfluidics and SPRi Engineering Research Center, Chongqing Medical University, Chongqing 400016, P. R. China

4. State Key Laboratory of Multi-phase Complex Systems, Institute of Process Engineering, Chinese Academy of Sciences, Beijing 100190, P. R. China

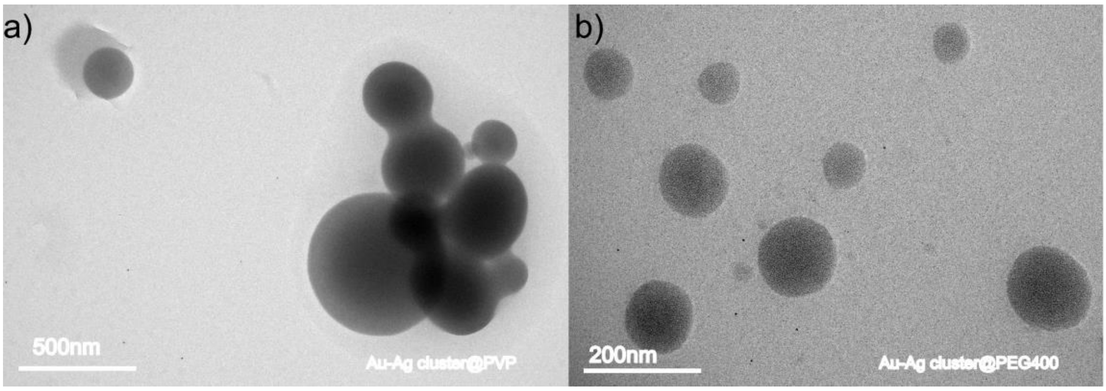

Figure S1. (a)TEM images of Au-Ag cluster @PVP. (b)TEM images of Au-Ag cluster @PEG400.

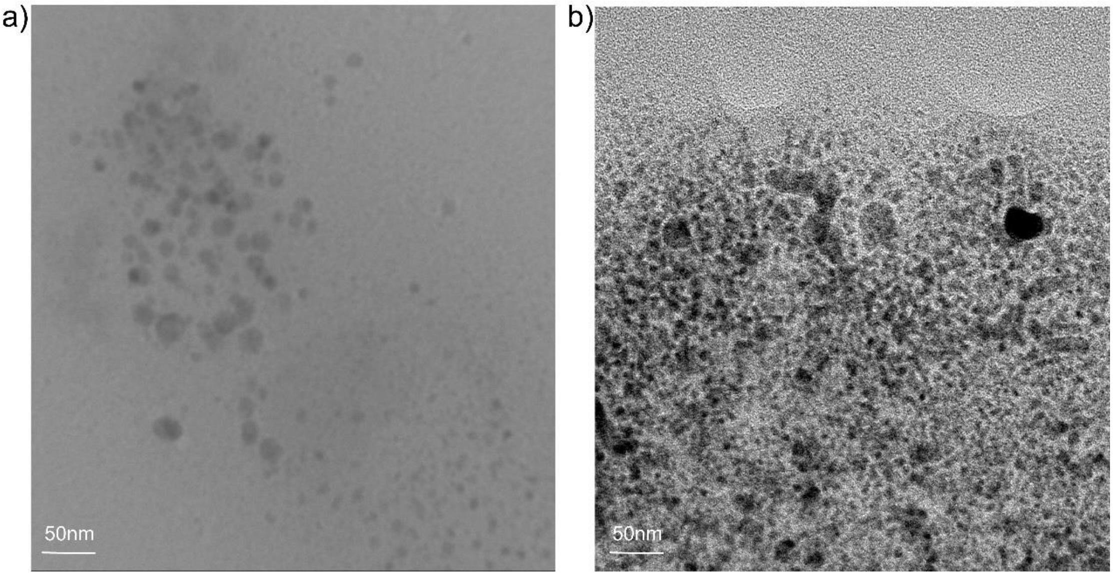

Figure S2. (a)TEM images of Au NCs. (b)TEM images of Ag NCs

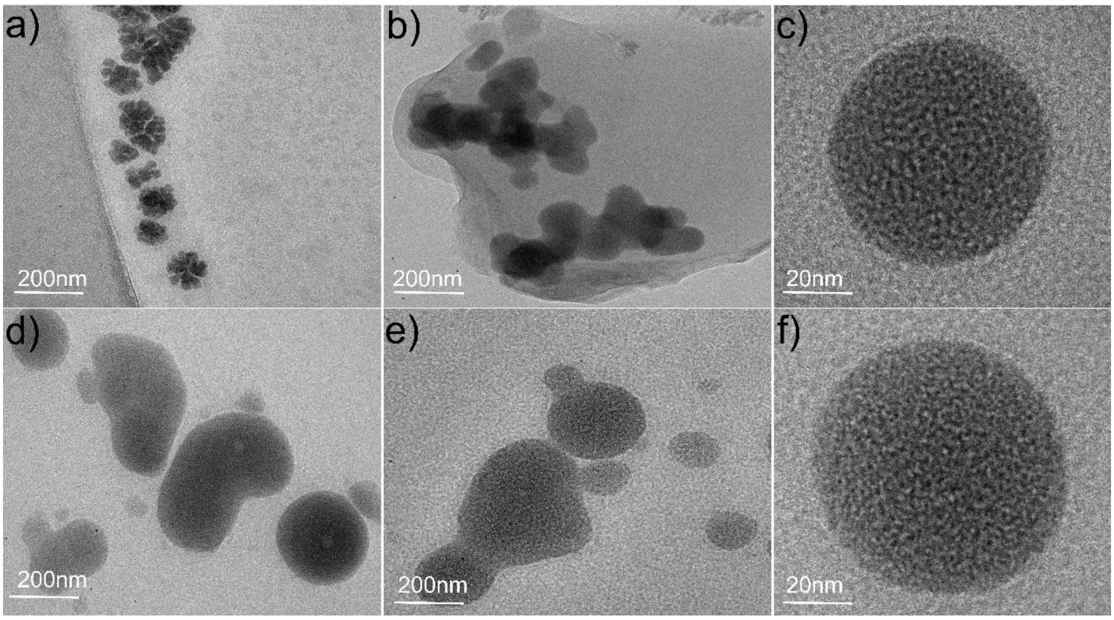

Figure S3. (a)and (b)TEM images of Au-Ag cluster @PVP. (c)HR-TEM images of Au-Ag cluster @PVP. (d)and (e)TEM images of Au-Ag cluster @PEG400. (f) HR-TEM images of Au-Ag cluster @PEG400.

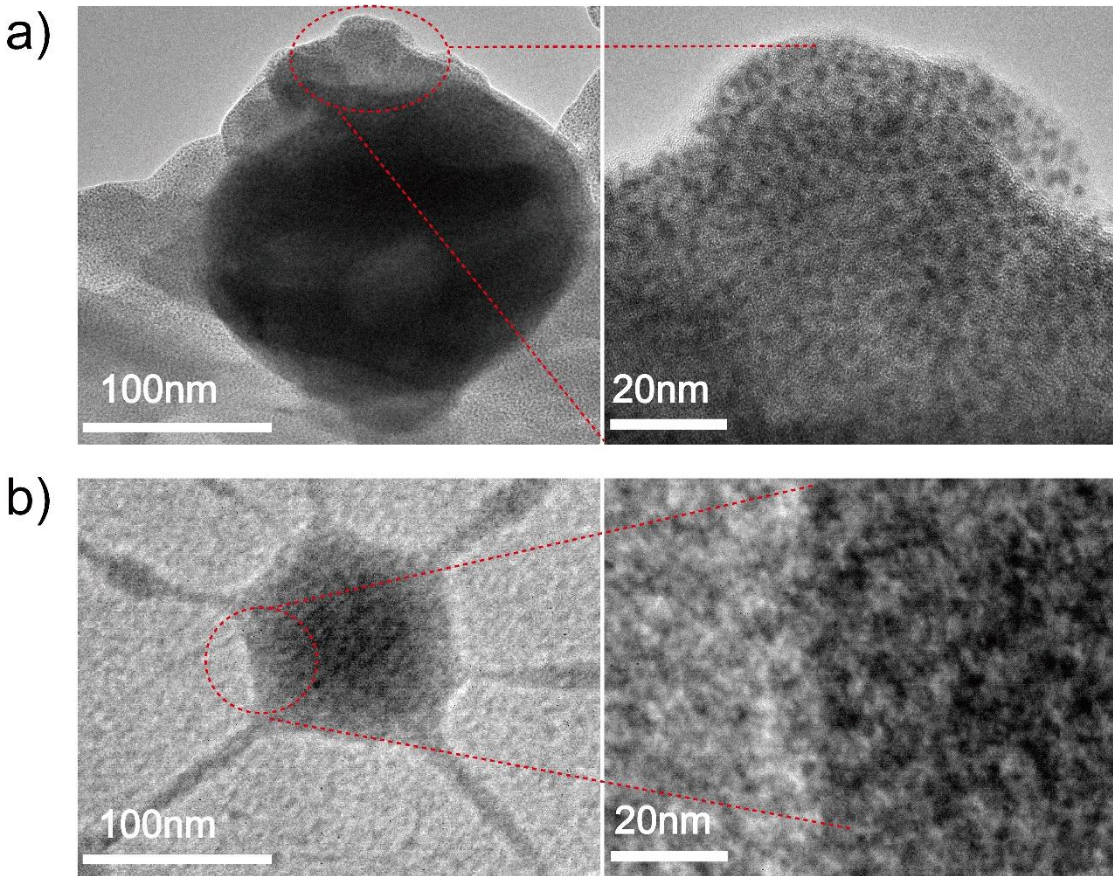

Figure S4. (a)HR-TEM images of Au-Ag cluster@PEG400-DOX; (b) HR-TEM images of Au-Ag cluster @PVP-DOX.

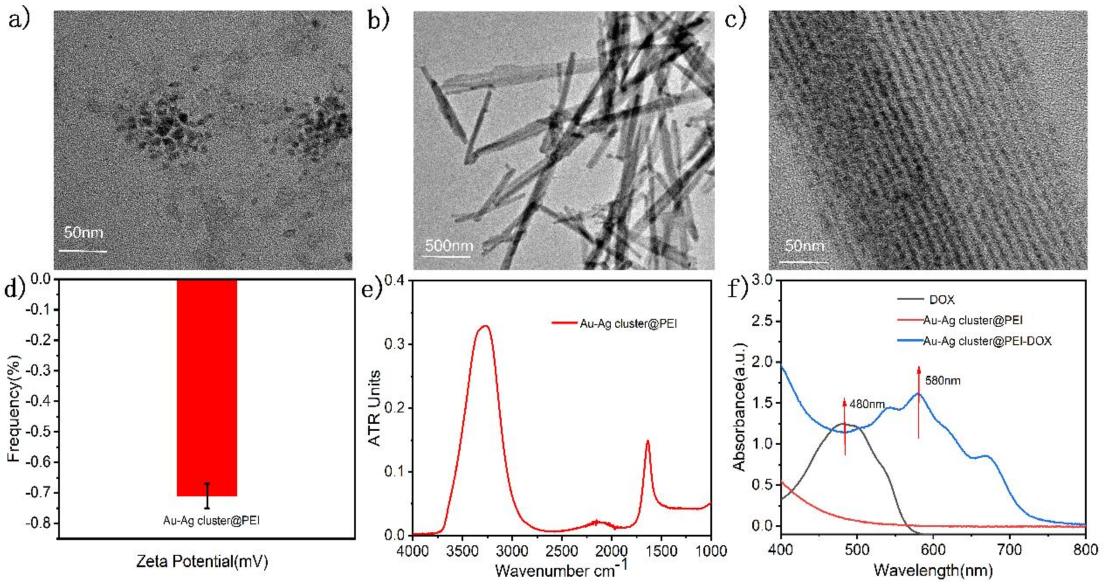

Figure S5. (a) TEM images of Au-Ag cluster @PEI NCs. (b) TEM images of Au-Ag cluster @PEI-DOX. (c)HR-TEM images of Au-Ag cluster @PEI-DOX. (d)Zeta potential of Au-Ag cluster @PEI. (e) ATR-FTIR spectra of Au-Ag cluster @PEI NCs. (f) UV–vis absorption of DOX、Au-Ag cluster @PEI and Au-Ag cluster @PEI-DOX .

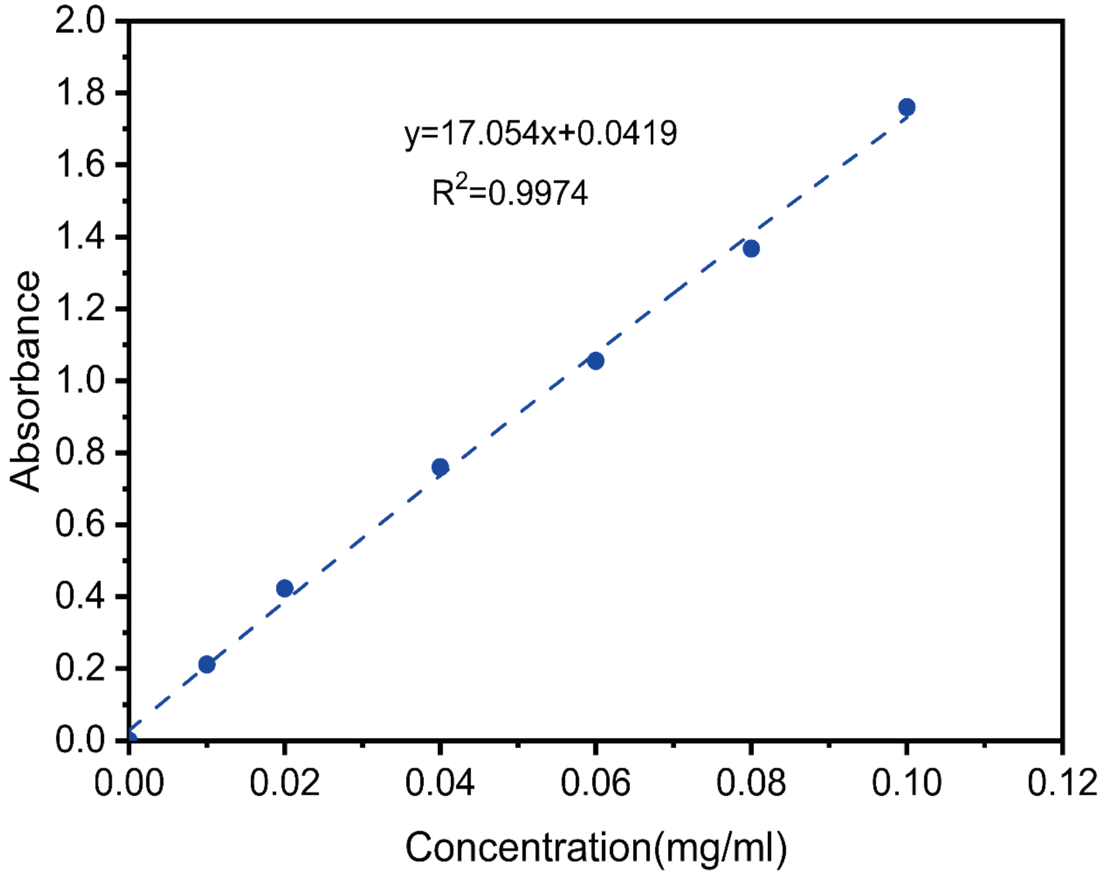

Figure S6. DOX standard curve for UV-Vis.

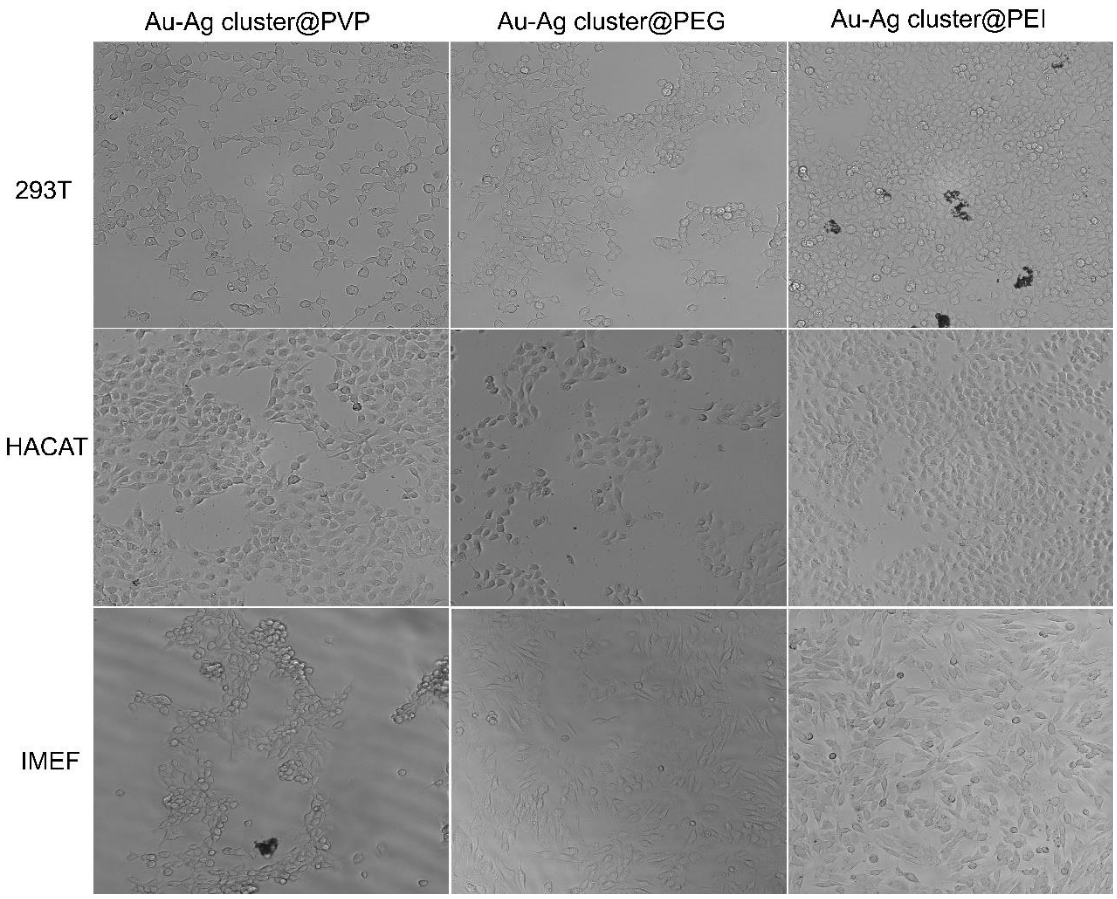

Figure S7. Representative Bright field microscope image of 293T, HACAT and IMEF cells, which were treated by using 50 μg ml^{-1} colloidal solutions (Au-Ag cluster @PVP, Au-Ag cluster @PEG400 and Au-Ag cluster @PEI) after 2 days, in preparation for biocompatibility measurements. The scale bar in the inset corresponds to 100 μm.

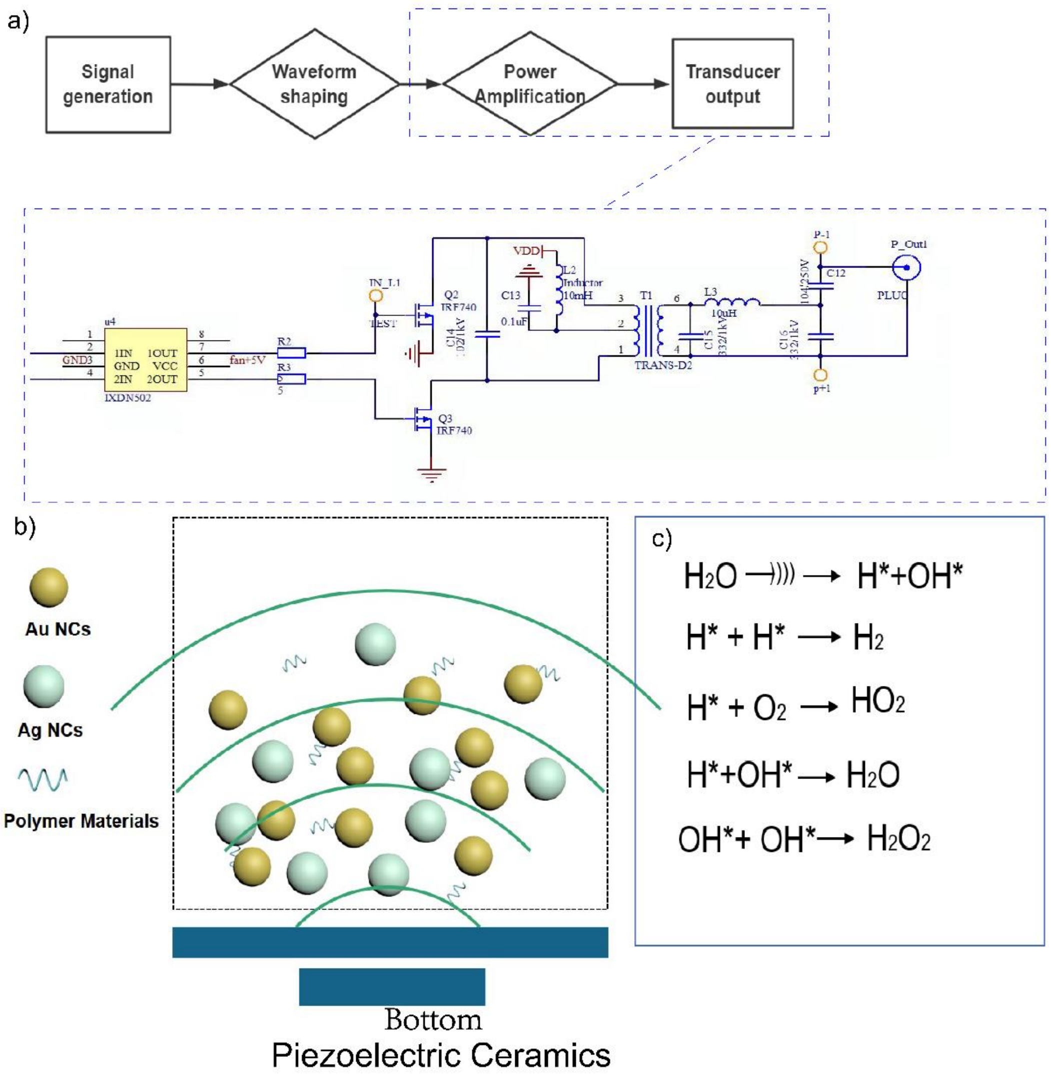

Figure S8. (a) Module and partial circuit diagram of the ultrasonic high frequency generator. (b) Profiles of nanoclusters moving back and forth in the ultrasonic trough under unidirectional emission of ultrasound. (c) Acoustic-chemical reactions present under ultrasonic treatment.

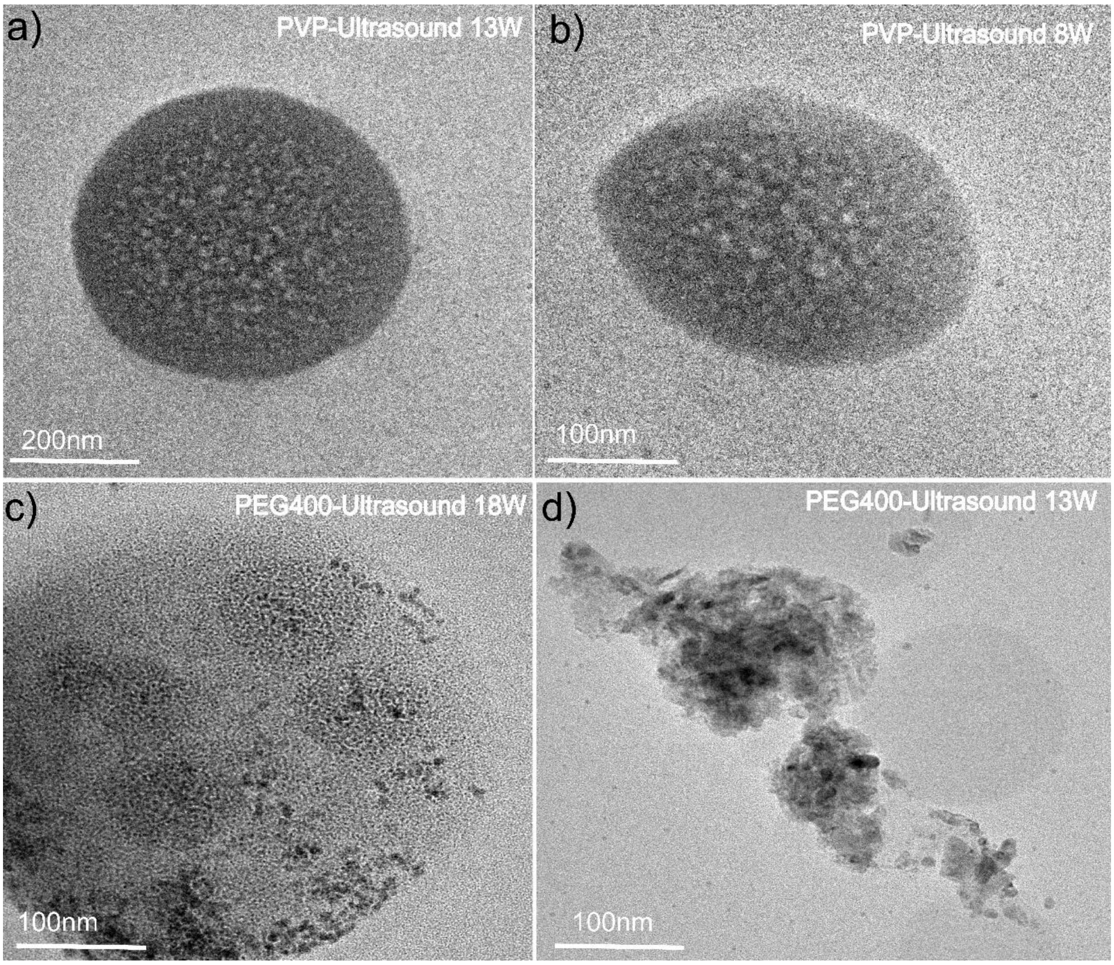

Figure S9. (a) TEM images of Au-Ag cluster @PVP prepared at 13W ultrasonic power. (b) TEM images of Au-Ag cluster @PVP prepared at 8W ultrasonic power. (c)TEM images of Au-Ag cluster @PEG400 prepared at 18W ultrasonic power. (d)TEM images of Au-Ag cluster @PEG400 prepared at 13W ultrasonic power.

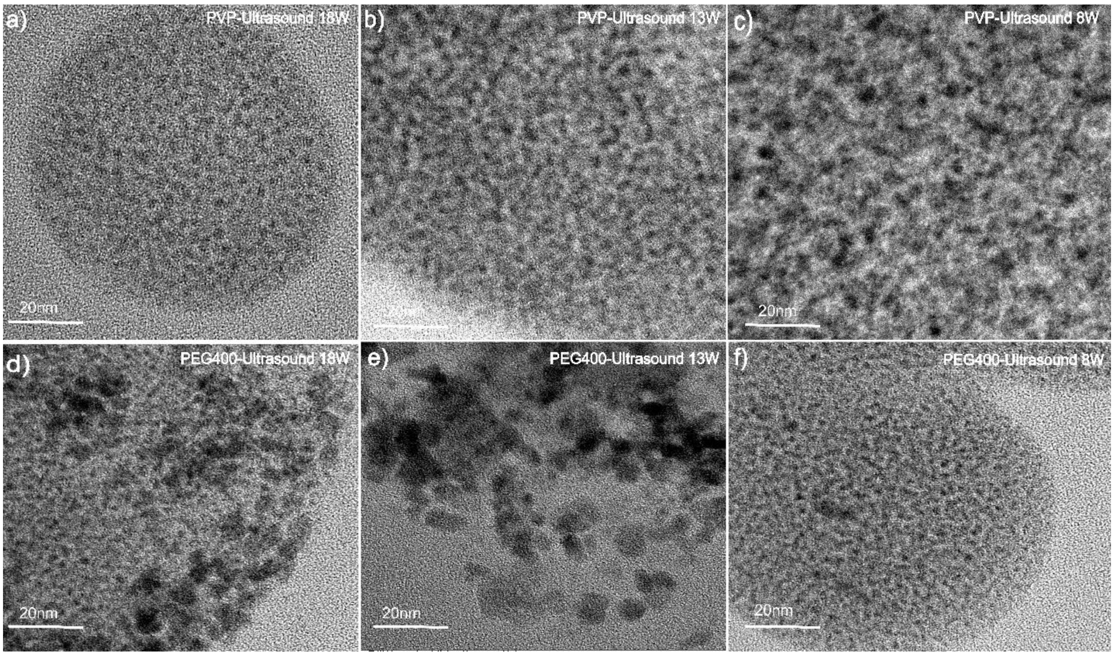

Figure S10. (a) (b) (c) High-resolution TEM images of Au-Ag cluster @PVP prepared at 18W, 13W, and 8W ultrasonic power. (d)(e)(f) High-resolution TEM images of Au-Ag cluster @PEG400 prepared at 18W, 13W, and 8W ultrasonic power.
